# Homozygous Nonsense *DSP* Variants Selectively Affecting Desmoplakin I Cause Carvajal Syndrome in a Consanguineous Arabian Family with Four Affected Female Siblings

**DOI:** 10.3390/jcm15155933

**Published:** 2026-07-29

**Authors:** Dalal A. Al-Mutairi, Mustafa A. Al-Qbandi, Ahmed AlTurki, Athbi A. Naief, Adam S. Helms

**Affiliations:** 1 Human Genetics Unit, Faculty of Medicine, Kuwait University, Kuwait City 13110, Kuwait; 2 Department of Pediatrics Cardiology, Chest Diseases Hospital, Al-Sabah Medical District, Kuwait City 70001, Kuwait; 3 Department of Medicine, Faculty of Medicine, Kuwait University, Kuwait City 13110, Kuwait; 4 Department of Radiology, Al Sabah Hospital, Kuwait City 13041, Kuwait; 5 Department of Internal Medicine, Division of Cardiovascular Medicine, University of Michigan, Ann Arbor, MI 48109, USA

**Keywords:** arrhythmogenic cardiomyopathy, *DSP* gene, consanguinity, epidermolytic palmoplantar keratoderma, sudden cardiac death

## Abstract

**Introduction:** Arrhythmogenic cardiomyopathy (ACM) is a myocardial disorder characterized by fibrofatty replacement of the myocardium and is associated with heart failure and sudden cardiac death. It can involve either ventricle, including left-dominant forms defined partly by genetic findings. **Methods:** We investigated a consanguineous family with four affected daughters presenting with ACM, epidermolytic palmoplantar keratoderma, and woolly hair. The two eldest siblings died suddenly during childhood. The remaining two affected siblings underwent genetic analysis using autozygosity mapping and whole-exome sequencing, followed by segregation analysis. **Results:** Autozygosity mapping identified a shared identity-by-descent (IBD) interval at the *DSP* locus on chromosome 6. Exome sequencing revealed a novel homozygous nonsense variant (c.4297C>T; p.Gln1433*; rs1554108283) in exon 23 of *DSP*. This variant introduces a premature termination codon within the region specific to transcript variant 1, sparing transcript variant 2. Both parents were heterozygous carriers. Cardiac imaging in the affected siblings demonstrated biventricular involvement, including left ventricular systolic dysfunction and right ventricular dilatation. Skin biopsy in one patient confirmed epidermolytic palmoplantar keratoderma. **Conclusions:** We describe a novel homozygous nonsense variant in the transcript 1-specific region of *DSP* causing Carvajal syndrome, characterized by severe biventricular ACM, epidermolytic palmoplantar keratoderma, and woolly hair, in a consanguineous Arabian family with four affected sisters.

## 1. Introduction

Desmoplakin (DSP) is the most abundant component of the desmosome and a member of the plakin superfamily. It is encoded by the *DSP* gene, which is highly expressed in the myocardium and skin epithelia. In addition, DSP plays a key role in the intestinal epithelium, where it anchors keratin intermediate filaments to desmosomes, thereby supporting mechanical resilience and maintaining barrier integrity [1]. DSP is essential for cell-to-cell adhesion junctions, anchoring intermediate filaments to desmosomes in both cardiomyocytes and skin [2,3].

Cardiac desmosomes are specialized intercellular junctions that provide strong mechanical coupling between adjacent cardiomyocytes. DSP is the terminal component of the desmosomal plaque and serves as the critical intracellular linker between desmosomal proteins and the intermediate filament network, which is predominantly composed of desmin in cardiac muscle. Disruption of this structural linkage compromises mechanical integrity and intracellular force transmission, predisposing the myocardium to fibrofatty replacement, ventricular dysfunction, and malignant arrhythmias.

At the cellular level, DSP serves as the essential intracellular linker that anchors desmosomal cadherin complexes to the intermediate filament network, thereby maintaining mechanical integrity and force transmission between adjacent cardiomyocytes. In cardiac tissue, the predominant intermediate filament protein is desmin (DES), which forms an extensive cytoskeletal scaffold connecting desmosomes, sarcomeres, mitochondria, and the nucleus [4,5,6].

Consequently, disruption of either DSP or DES compromises the structural continuity of the cardiomyocyte and increases susceptibility to mechanical stress, ultimately leading to myocardial injury and arrhythmogenesis. This functional relationship is further supported by the remarkable phenotypic overlap between DSP- and DES-related cardiomyopathies. Notably, the DES missense variants p.R127P and p.Glu401Asp have been shown to impair desmin filament assembly, disrupt cellular architecture and membrane integrity, and cause severe arrhythmogenic cardiomyopathy, including left-dominant disease with high cardiac morbidity and mortality [4,5].

These observations reinforce the concept that defects affecting either the desmosomal plaque or its intermediate filament attachment converge on a common pathogenic pathway, highlighting the critical role of the DSP- DES axis in preserving cardiomyocyte integrity and normal cardiac function. Accordingly, pathogenic variants in both *DSP* and *DES* can produce overlapping arrhythmogenic cardiomyopathy phenotypes, highlighting the functional importance of the desmosome-intermediate filament complex [5,7,8,9].

The *DSP* gene encodes two major isoforms generated by alternative splicing: a long isoform (DSP-I) and a short isoform (DSP-II). The two isoforms are identical in the N- and C-terminal domains; however, DSP-II contains only one-third of the rod domain due to alternative splicing involving exon 23 [10,11]. Pathogenic variants in *DSP* are associated with a spectrum of inherited diseases, incuding arrhythmogenic cardiomyopathy (ACM), Carvajal syndrome, recessive cardiocutaneous syndromes, palmoplantar keratoderma, and woolly hair [12,13,14,15,16].

Carvajal syndrome is a rare autosomal recessive cardiocutaneous disorder characterized by arrhythmogenic cardiomyopathy, woolly hair, and palmoplantar keratoderma. It is closely related to Naxos disease, which presents with a similar clinical phenotype; however, the two disorders are distinguished by their molecular basis. Carvajal syndrome is caused by biallelic pathogenic variants in *DSP*, whereas Naxos disease results from biallelic pathogenic variants in *JUP*. Although considerable phenotypic overlap exists, Carvajal syndrome more commonly demonstrates predominant left ventricular or biventricular involvement [12,17].

Arrhythmogenic cardiomyopathy (ACM) is a genetically heterogeneous inherited myocardial disease characterized by progressive myocardial degeneration, ventricular arrhythmias, heart failure, and an increased risk of sudden cardiac death. Pathogenic variants have been identified in more than 25 genes, most of which encode proteins involved in the cardiac desmosome, including *PKP2*, *DSP*, *DSG2*, *DSC2*, and *JUP*. Although non-desmosomal genes have also been implicated, desmosomal gene defects account for the majority of genetically confirmed ACM cases, underscoring the essential role of intercellular adhesion in maintaining myocardial integrity and electrical stability [8,18,19].

The association between *DSP* pathogenic variants and hereditary cardiovascular disease was first reported in 2002 in an Italian family with arrhythmogenic cardiomyopathy (ACM) carrying missense variants in *DSP* [20]. Subsequently, in a cohort of 38 ACM patients, four were found to carry different *DSP* pathogenic variants [21]. The main clinical features included a high rate of sudden cardiac death (SCD) and left ventricular (LV) involvement. In addition, two patients presented with chest pain, ST-segment elevation on electrocardiogram (ECG), and myocardial enzyme release despite angiographically normal coronary arteries [19,21]. Another study of 200 ACM patients using cardiac magnetic resonance (CMR) imaging demonstrated a high incidence of LV involvement (84%) in individuals with *DSP* variants, consistent with a left-dominant ACM phenotype [22]. These patients also exhibited a higher burden of ventricular arrhythmias (VA), exceeding the degree of systolic dysfunction. Remarkably, myocarditis-like episodes, referred to as “hot phases,” have also been described [19,22].

Patients with ACM display a wide spectrum of right ventricular (RV) and left ventricular (LV) morpho-functional abnormalities. Currently, three phenotypes are recognized: the classical right-dominant form of arrhythmogenic right ventricular cardiomyopathy (ARVC), with absent or minimal LV involvement; the biventricular (BIV) form, involving both ventricles; and the left-dominant variant, termed arrhythmogenic left ventricular cardiomyopathy (ALVC), characterized by minimal RV abnormalities [18,19,22]. Importantly, 57% of ALVC individuals carry pathogenic variants in the *DSP* gene [2].

Distinguishing ALVC from dilated cardiomyopathy (DCM) can be challenging [23,24]. For example, in broad cohorts, patients diagnosed with DCM may harbour desmosomal variants associated with ACM [25]. A key feature of ACM is myocardial fibrosis, predominantly involving the subepicardial layer of the inferolateral left ventricular segments [26]. In ALVC, fibrosis is a primary disease process and precedes systolic dysfunction, whereas in DCM, it is secondary to ventricular dilatation [26,27]. Autopsy studies of ALVC patients with *DSP* variants demonstrate fibrofatty myocardial infiltration of the left ventricle, which is considered the primary substrate for clinical arrhythmias [28]. Genotype–phenotype correlation studies further showed that *DSP* pathogenic variants are mainly associated with LV structural involvement, high arrhythmic risk, and ventricular dysfunction [29]. In addition, DCM patients who are heterozygous carriers of *DSP* variants exhibit a more arrhythmogenic phenotype, even in the absence of LV dilatation or systolic dysfunction [23,24]. A multicenter study of 183 DCM heterozygous carriers (mostly *DSP*) reported a high incidence of life-threatening ventricular arrhythmias, including in patients with only mildly reduced left ventricular ejection fraction (LVEF), along with increased sudden cardiac death risk [30]. Taken together, the clinical phenotype associated with *DSP* pathogenic variants is referred to as “desmoplakin cardiomyopathy,” a distinct form of ACM characterized by episodes of acute myocardial injury, high arrhythmic risk, extensive fibrosis, and frequent left ventricular involvement [31,32,33].

## 2. Methods

### 2.1. Human Subjects

**Figure 1:**
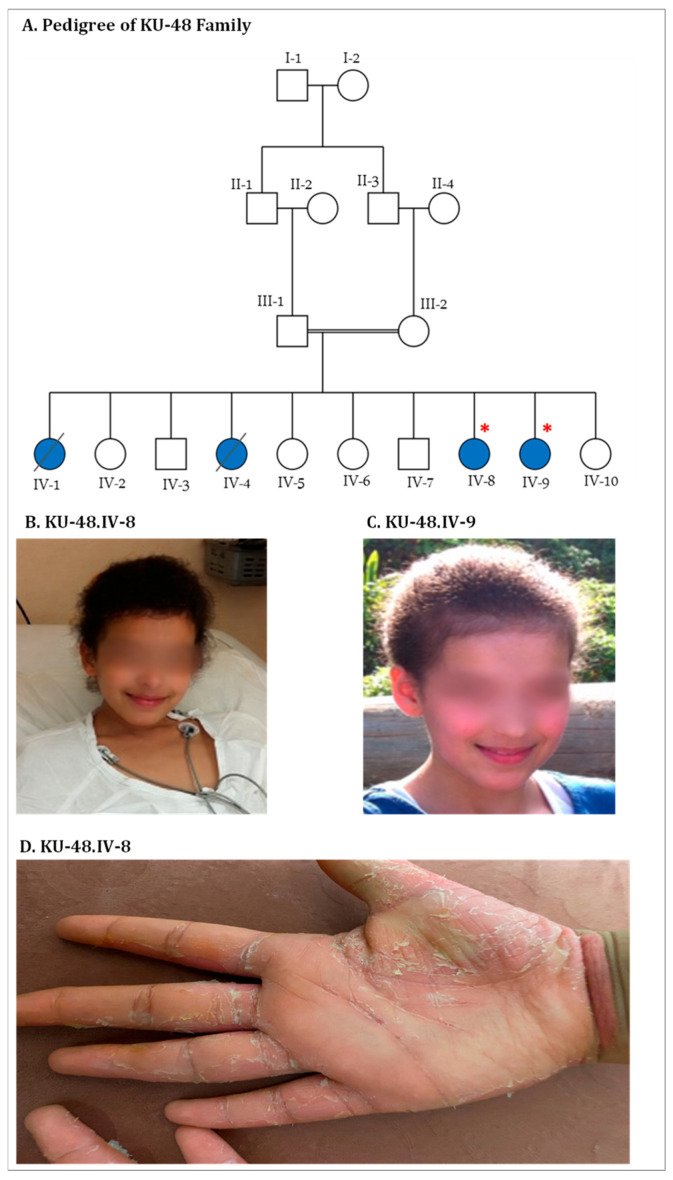
Pedigree and the clinical features of Carvajal syndrome for Family KU-48. (**A**): The pedigree of Family KU-48, which has a total of four affected females with Carvajal syndrome. The genetic studies were done on the two affected female siblings (KU-48.IV-8 and KU-48.IV-9) highlighted by red asterisks. (**B**,**C**): Photographs of the two affected siblings, KU-48.IV-8 and KU-48.IV-9, respectively, showing that both have woolly hair. (**D**): Striate pattern of palmoplantar hyperkeratosis in patient KU-48.IV-8. The photograph was taken during chemical peeling, which accentuates the hyperkeratotic lesions. The patient’s hand showed focal hyperkeratosis on the palm with relative sparing of parts of the pressure-bearing sites.

We describe a multiplex KU-48 family with four affected daughters presenting with autosomal recessive arrhythmogenic cardiomyopathy with biventricular involvement, epidermolytic palmoplantar keratoderma, and woolly hair. The patients exhibited a severe phenotype with early onset and rapid progression to heart failure. Ethical approval for this study was obtained from the Kuwait Ministry of Health Research Ethics Committee (Ethics ID: 62/2013). Written informed consent was obtained from adult participants (III-1, III-2, IV-2, IV-3, IV-5, IV-6, and IV-7) and from the parents of minor participants (IV-8, IV-9, and IV-10). Blood samples were collected from affected individuals, unaffected siblings, and parents (Figure 1A). Clinical data, including cardiac magnetic resonance (CMR), electrocardiograms (ECGs), and echocardiography, were obtained for the two available affected patients (IV-8 and IV-9) to correlate genotype with phenotype. A clinical examination revealed woolly hair in both affected siblings, as shown in Figure 1B,C. We demonstrated the characteristic striate palmoplantar hyperkeratosis in patient KU-48.IV-8 (Figure 1D), although this cutaneous feature was exhibited by both affected siblings. Representative electrocardiograms illustrating the characteristic cardiac conduction abnormalities observed in the affected siblings are presented in the Supplementary Materials (Figure S1).

### 2.2. Genomic DNA and Exome Sequencing

Whole-exome sequencing was performed on genomic DNA extracted from peripheral blood of the two affected individuals (IV-8 and IV-9, Figure 1A). DNA quality and concentration were assessed prior to library preparation. Target enrichment of coding regions was performed using the SureSelect Human All Exon v6 capture kit (Agilent Technologies, Santa Clara, CA, USA) according to the manufacturer’s protocol. Briefly, genomic DNA was fragmented, ligated to sequencing adapters, and hybridized to biotinylated RNA probes for exome capture, followed by PCR amplification of the enriched libraries.

Sequencing was carried out on an Illumina HiSeq 2500 platform (Illumina, San Diego, CA, USA) using paired-end sequencing chemistry. Raw reads were processed through standard Illumina pipelines, including base calling, demultiplexing, and quality filtering. Reads were aligned to the human reference genome (hg19), and standard post-alignment processing and variant calling were performed. Generated variant call format (VCF) files were used for downstream analyses, including autozygosity mapping, identity-by-descent (IBD) analysis, and variant prioritization under a recessive inheritance model.

### 2.3. Autozygosity Mapping and Variant Screening

**Figure 2:**
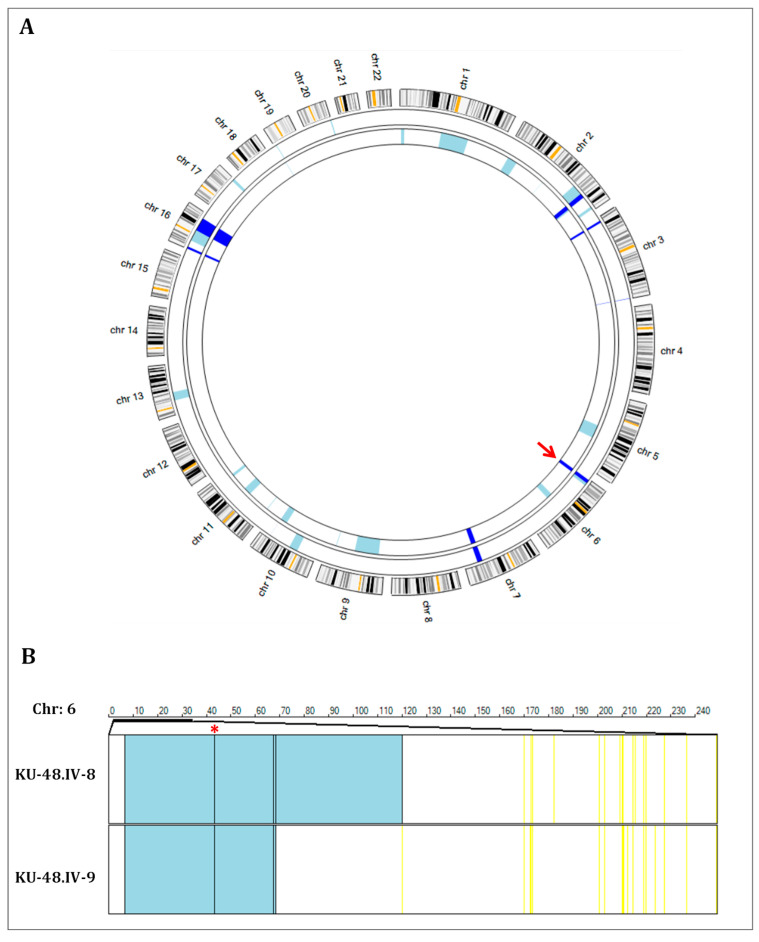
Genetic linkage of 22 autosomal chromosomes of Family KU-48. (**A**): AgileMultiIdeogram for the two affected female siblings. The ideogram is representing the linkage scan as a circular axis for the KU-48.IV-8 and KU-48.IV-9 individuals belonging to the same family in one group. In multiplex families, the exclusive regions of homozygosity (ROH) shared between the affected relatives are displayed as navy-blue bars. The homozygous regions that are not shared among affected relatives are displayed as light blue bars. As seen from the initial linkage scan, the two patients have shared IBD intervals (navy-blue bars) at chr:2, chr: 6, chr:7, and chr:16. Screening the exome data at the shared IBD interval led to mapping of the novel *DSP* variant to a significant, shared IBD interval in chr: 6 (red arrow). In the ideogram, the linkage scan is presented from the ideogram edge (first circus: KU-48.IV-8) towards the centre (last circus: KU-48.IV-9). (**B**): Multiple linkage analysis at chr: 6 for the two affected siblings was performed using AgileVCFMapper software. The shared IBD interval at chr: 6 was estimated to be 2,774,723–10,721,339. The physical location of *DSP* is at Chr6: 7,541,671–7,586,714 (within the estimated IBD interval) and is highlighted by the red asterisk. As seen, each row represents the genotypes for chr: 6 for one affected individual. The homozygous discordant alleles are represented as yellow lines; concordant autozygous alleles are represented as blue lines; and rare concordant autozygous alleles most likely harbouring rare pathogenic variants are represented as black lines.

Genetic screening using autozygosity mapping was performed as previously described [34]. Initially, AgileMultiIdeogram software (version 3) was used to visualize homozygous intervals derived from exome data, in which all regions of homozygosity were displayed on a circular ideogram representing the 22 autosomal chromosomes in the affected individuals, as shown in Figure 2A [34]. Genome annotation was based on the Human Genome Build hg19 (UCSC Genome Browser).

Subsequently, linkage analysis was performed in the two affected ACM siblings (IV-8 and IV-9) using AgileVCFMapper software (version 3), an Autozygous Variant Viewer designed to identify identity-by-descent (IBD) intervals across chromosomes using exome-derived genotypes [35]. This approach enabled the reconstruction of shared haplotypes and the identification of overlapping regions of autozygosity. These shared homozygous and concordant variants were used to define candidate founder intervals harbouring the disease-causing variant, as illustrated in Figure 2B and Figure S2.

### 2.4. Variant Confirmation and Segregation Analysis

Primers for variant confirmation and segregation analysis were designed using Primer3 software (https://primer3.ut.ee/; accessed on 15 June 2025). Exon sequences were retrieved from the University of California Santa Cruz Genome Browser (http://genome.ucsc.edu/; accessed on 30 June 2025) for exon 23 of the *DSP* gene. For the novel founder nonsense variant rs1554108283 (RefSNP Report, dbSNP, NCBI), the forward primer sequence was 5′-GAGACCGAGATCAACATCACC-3′ and the reverse primer sequence was 5′-CTCCGTCGCACTACTGTTTG-3′, which were used to amplify and sequence the homozygous variant identified in the two affected female siblings with ACM. In addition, all available family members were screened for this variant; parental samples were sequenced to confirm segregation with the disease phenotype, and six unaffected siblings were analyzed to determine heterozygous carrier status within the extended family. Sequencing reactions were performed as previously described [36], and Sanger sequencing results were analyzed using GeneScreen software (version 3) [37].

### 2.5. Histopathology Examination of a Skin Biopsy

Written informed consent was obtained from the patient’s parents. Skin biopsy was performed under local anesthesia. Specimens were fixed in buffered formalin, processed routinely, and stained with hematoxylin and eosin. A consultant dermatopathologist examined the slides using light microscopy [38]. Desmoplakin immunohistochemistry was not performed on the skin biopsy, as this test is not available at our institution. The diagnosis was instead established through genetic testing, which identified a pathogenic variant consistent with the clinical and histological findings.

### 2.6. In Silico Pathogenicity Prediction and ACMG/AMP Variant Classification

The pathogenicity of the identified DSP variant was evaluated using multiple in silico prediction tools and publicly available variant annotation databases. Computational evidence was integrated with population frequency, segregation data, predicted molecular consequence, gene- and disease-specific evidence, and the published literature to classify the variant according to the American College of Medical Genetics and Genomics/Association for Molecular Pathology (ACMG/AMP) guidelines and ClinGen Sequence Variant Interpretation (SVI) Working Group recommendations. 

The *DSP* c.4297C>T (p.Gln1433*) variant was evaluated using a panel of complementary computational prediction algorithms that assess variant pathogenicity, functional impact, and evolutionary conservation, including MutationTaster, CADD, FATHMM-MKL, Eigen-PC, DANN, fitCons, and LRT [39,40,41,42,43,44,45]. A detailed description of the computational analyses, together with the complete prediction results and evidence used for variant interpretation, is provided in the Supplementary Material and Supplementary Table S1.

## 3. Results

### 3.1. Medical History of the Two Patients Under Study

**Figure 3:**
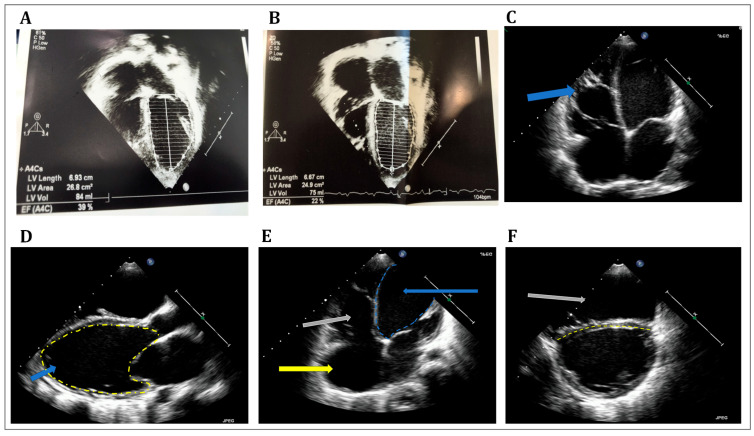
Echocardiography of affected siblings. (**A**) KU-48.IV-8 shows moderate left ventricular systolic dysfunction. (**B**) KU-48.IV-9 shows severe left ventricular systolic dysfunction. (**C**) KU-48.IV-8 shows dilated left ventricle in parasternal long axis view. (**D**) KU-48.IV-8 shows trabeculated right ventricle (blue arrow) in apical four-chamber view. (**E**) KU-48.IV-8 shows biventricular (blue and grey arrows) and right atrial dilatation (yellow). (**F**) KU-48.IV-8 shows right ventricular dilatation (grey arrow) with septal bowing.

According to medical records, patient KU.48.IV-8 developed initial symptoms at the age of six years, including shortness of breath and palpitations. She was further evaluated due to a family history of sudden cardiac death in two older sisters at the ages of 8 and 9 years. Baseline electrocardiogram showed incomplete right bundle branch block (Figure S1A). She was initially diagnosed with arrhythmogenic cardiomyopathy (ACM) (Figure 3A), with evidence of left ventricular dilatation (Figure 3C) and right ventricular trabeculation (Figure 3D), and was started on neurohormonal therapy. During follow-up, she experienced recurrent episodes of chest pain and dyspnea. An implantable cardioverter-defibrillator (ICD) was later implanted; by that time, echocardiography showed disease progression with a left ventricular ejection fraction (LVEF) of 20%, biventricular dilatation (Figure 3E), right ventricular strain (Figure 3F), and secondary mitral and tricuspid regurgitation. Her heart failure progressed at the age of 11, requiring inotropic support, and she subsequently suffered cardiac arrest with no return of spontaneous circulation while awaiting cardiac transplantation.

**Figure 4:**
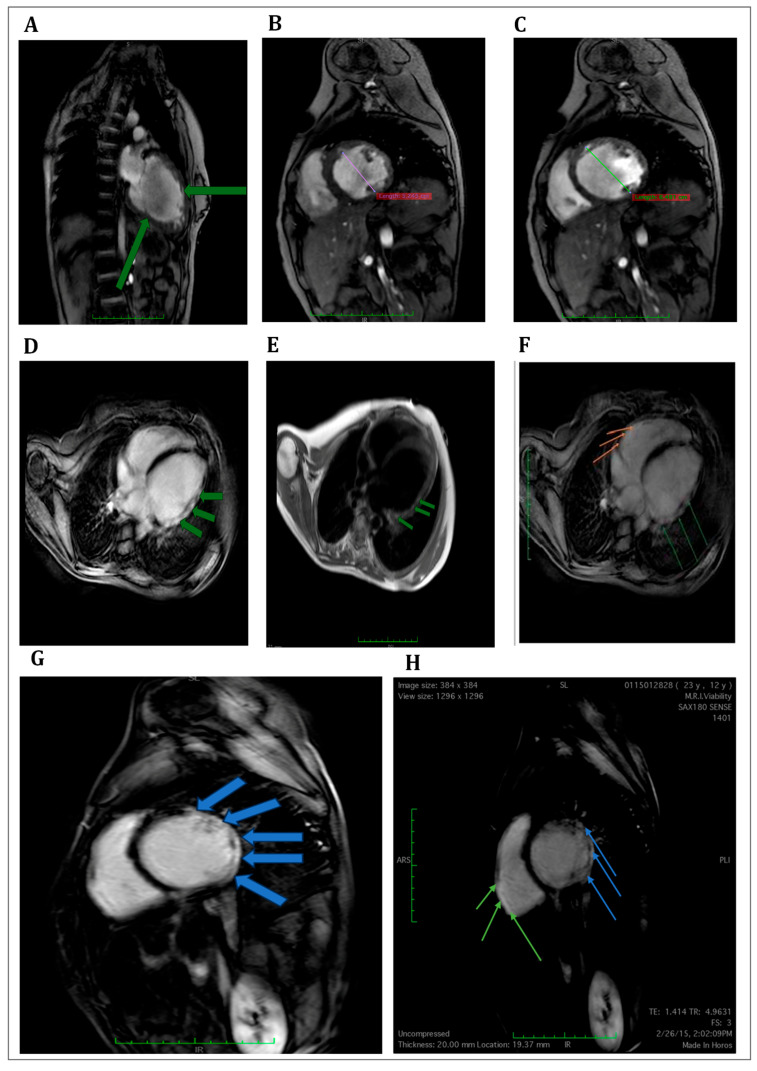
Cardiac MRI (KU-48.IV-9). (**A**) Marked left ventricular dilatation (green arrow). (**B**) End-systolic LV diameter of 5.24 cm (red line). (**C**) End-diastolic LV diameter of 6.48 cm (green line). (**D**) Subepicardial late gadolinium enhancement in the lateral LV wall on axial imaging. (**E**) Dark-blood axial imaging showing fatty infiltration in the same region of the lateral LV wall. (**F**) Axial images showing late gadolinium enhancement at the lateral wall of the left ventricle (green arrows) and involvement of the right ventricular wall (orange arrows) indicating fibrotic changes. (**G**) Two-chamber view: Late gadolinium enhancement at the lateral wall of the left ventricle (blue arrows) indicating fibrotic changes. (**H**) Two-chamber view: Late gadolinium enhancement at the anterior, lateral, and inferior walls of the left ventricle (green arrows) and involvement of the right ventricular wall (green arrows) indicating fibrotic changes.

Patient KU.48.IV-9, two years younger than KU.48.IV-8, had a normal screening echocardiogram at the age of 5 years; however, she demonstrated progressive left ventricular dilatation and declining LVEF during follow-up (Figure 3B). She also developed recurrent episodes of dyspnea and occasional chest pain. Baseline electrocardiogram showed sinus rhythm with frequent premature ventricular complexes (PVCs) and an epsilon wave; the PR interval was also prolonged at 200ms and the QRS voltages were low borderline in the precordial leads (Figure S1B). She was not on any cardiac medication at the time of the electrocardiogram. At the age of 12 years, an ICD was implanted, and she received an appropriate shock several months later. Cardiac magnetic resonance imaging performed prior to ICD implantation demonstrated a dilated left ventricle (LVEDV 165 mL, LVESV 146 mL) with a severely reduced LVEF of 10% (Figure 4A–C), global hypokinesia, and an apical thrombus. The right ventricle was also dilated (RVEDV 106 mL, RVESV 79 mL) with an RVEF of 25%. Late gadolinium enhancement revealed diffuse subepicardial enhancement involving nearly the entire left ventricular lateral wall (Figure 4D–F) and the right ventricular apex (Figure 4F). Despite treatment with amiodarone, she experienced clinical deterioration with worsening heart failure and ventricular arrhythmias. She ultimately suffered cardiac arrest with a non-shockable rhythm and no return of spontaneous circulation. Cardiac magnetic resonance cine imaging further demonstrated severe right ventricular systolic dysfunction with regional wall motion abnormalities (Supplementary Video S1). Late gadolinium enhancement demonstrated fibrotic changes involving the left ventricular myocardium and the right ventricular wall, as illustrated in the two-chamber cardiac magnetic resonance images (Figure 4G,H). Clinical, electrocardiographic, echocardiographic, and cardiac MRI findings in the two affected siblings (KU-48.IV-8 and KU-48.IV-9) are presented in Table 1.

**Figure 5:**
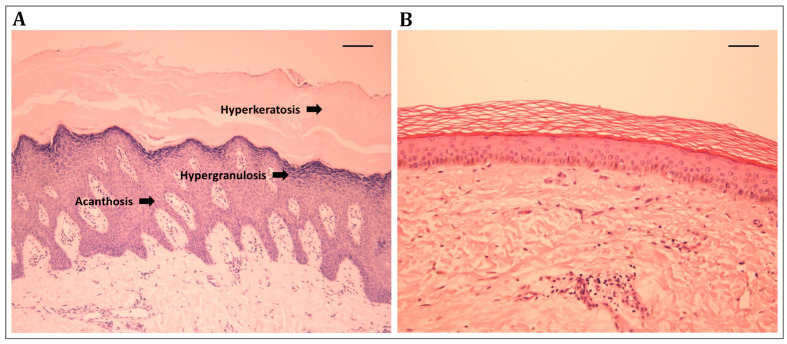
Palmoplantar keratoderma skin biopsy findings. (**A**) Patient KU-48.IV-8 shows epidermal hyperkeratosis, hypergranulosis, and acanthosis with elongated rete ridges. (**B**) Normal skin for comparison. A 4 mm skin biopsy was obtained from the hypothenar region of the palm. Scale bar is 20 µm.

Histologic examination of a hematoxylin and eosin-stained skin biopsy from a keratoderma lesion in patient KU-48.IV-8 at 10 years of age revealed hyperkeratosis, hypergranulosis, irregular acanthosis, and degenerative keratinocytes, with a focal band-like lymphohistiocytic infiltrate in the papillary dermis, as shown in Figure 5.

### 3.2. Autozygosity Mapping Reveals a Novel Founder Homozygous Nonsense Variant in DSP

Patients with arrhythmogenic cardiomyopathy (ACM) are referred to the Ministry of Health Chest Diseases Hospital in Kuwait for genetic evaluation. The KU-48 family was suspected to have a hereditary disorder based on the presence of four affected female siblings with an identical clinical phenotype. This extended consanguineous family comprises seven daughters and three sons, as shown in the pedigree (Figure 1A). Four female siblings (IV-1, IV-4, IV-8, and IV-9) were affected with arrhythmogenic cardiomyopathy (ACM) involving both ventricles, epidermolytic palmoplantar keratoderma, and woolly hair (Figure 1B–D).

Two affected individuals (IV-1 and IV-4) died at the ages of 10 years and 12 years. DNA samples were obtained from the two surviving affected siblings (IV-8 and IV-9), respectively. DNA was subsequently collected from six unaffected siblings (IV-2, IV-3, IV-5, IV-6, IV-7, and IV-10) and both parents (III-1 and III-2). Whole-exome sequencing was performed on the two surviving affected individuals (IV-8 and IV-9).

Autozygosity mapping was applied in two analytical stages to identify shared homozygous regions. Initial genome-wide linkage analysis was performed in the two affected siblings jointly, revealing shared identity-by-descent (IBD) intervals across multiple autosomal chromosomes (Figure 2A). In consanguineous families, these shared autozygous segments represent critical regions for identifying recessive disease-causing variants [34,46]. The width of each IBD peak reflects the extent of homozygosity across the genome.

Shared IBD intervals were identified across chromosomes 2, 3, 6, 7, and 16 and are summarized in Table 2. Genomic coordinates are based on the GRCh38 reference genome, with interval boundaries defined by start and end positions, and size calculated in megabases (Mb). These regions represent candidate loci potentially harbouring the disease-causing variant.

Further linkage refinement using autozygosity mapping confirmed concordant homozygous segments on chromosomes 2, 3, 7, and 16 (Figure S2), which were filtered for rare homozygous variants but did not reveal a pathogenic candidate. In contrast, the shared IBD interval on chromosome 6 contained a novel nonsense variant in *DSP* (Figure 2B), strongly implicating this locus as the disease-causing region.

The KU-48 family demonstrated a consistent run of homozygosity (ROH) spanning the *DSP* locus on chromosome 6 (highlighted by a red arrow in Figure 2A). High-resolution linkage analysis identified a distinct shared autozygous interval of approximately 8 Mb (chr6: 2,720,193–10,829,087) in the two affected individuals (Figure 2B). This interval was detectable only through high-resolution SNP-based analysis of exome data using AgileVCFMapper. The *DSP* gene is located within this region (chr6: 7,541,617–7,586,714), marked by a red asterisk in Figure 2B. This helps to prioritize the homozygous pathogenic variant among nearly 200 shared homozygous variants identified in the two available siblings. All variants located within shared IBD segments across chromosomes 2, 3, 6, 7, and 16 were systematically evaluated for pathogenicity to exclude alternative candidate genes (Figure S2). These analyses supported *DSP* as the most likely disease-causing gene in this family.

**Figure 6:**
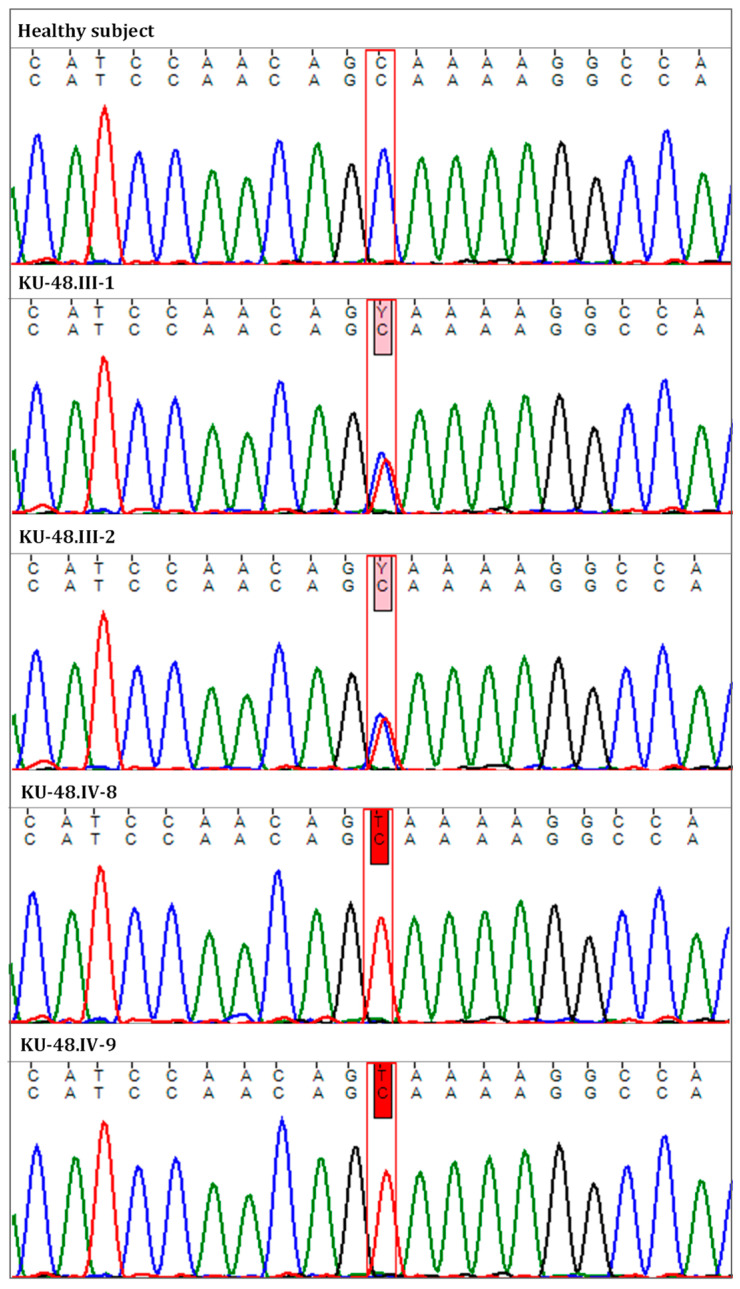
*DSP* variant chromatographs for the two affected siblings and the parents of Family KU-48. Chromatogram sequences for the healthy subject, the parents (KU-48.II-1 and KU-48.II-2), and the two ACM patients (KU-48.III-1 and the KU-48.III-2) are shown. The two affected siblings, KU-48.IV-8 and KU-48.IV-9, carry the homozygous nonsense variant (c.4297C>T; p.Gln1433*; rs1554108283) in exon 23 of *DSP* that results in a premature termination codon. Segregation analysis demonstrates that the parents, KU-48.III-1 and KU-48.III-2, are heterozygous carriers for this variant. At the pathogenic variant locus, pink rectangles indicate heterozygous variants, whereas red rectangles indicate homozygous pathogenic variants. The two red lines indicate the nucleotide position that differs from the wild-type allele.

Detailed variant screening of the chromosome 6 IBD region revealed a novel homozygous nonsense variant (c.4297C>T; p.Gln1433*; rs1554108283) in exon 23 of *DSP*. This loss-of-function variant introduces a premature termination codon and is classified as pathogenic according to ACMG guidelines. Sanger sequencing confirmed that both affected individuals carry the same homozygous variant (Figure 6). Segregation analysis demonstrated that the variant co-segregates with the disease phenotype in the family. Both parents were confirmed as heterozygous carriers (Figure 6), supporting autosomal recessive inheritance and confirming this variant as the causal mutation underlying Carvajal syndrome in the four affected female siblings.

**Figure 7:**
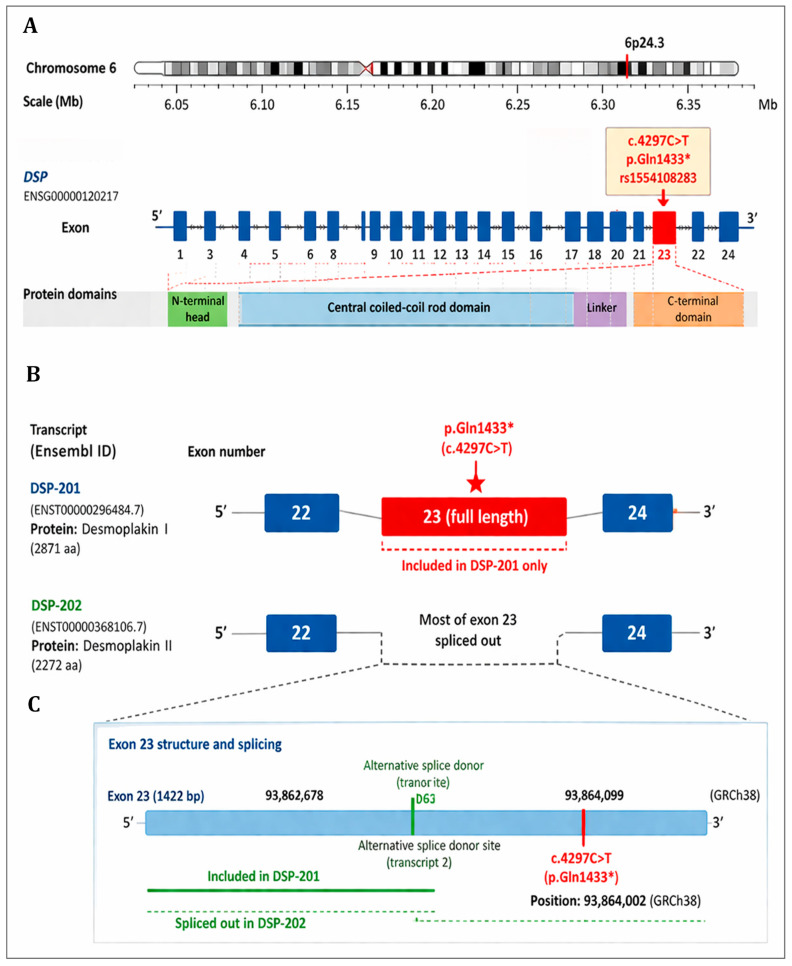
Gene structure, transcript specificity, and predicted impact of the *DSP* c.4297C>T (p.Gln1433*) variant. (**A**) Schematic representation of the *DSP* gene located on chromosome 6p24.3, showing the exon organization and corresponding protein domains. The homozygous nonsense variant c.4297C>T (p.Gln1433*) is located in exon 23, which is unique to the DSP-201 transcript. (**B**) Comparison of the two major DSP transcript isoforms. The c.4297C>T (p.Gln1433*) variant is present within exon 23 of the full-length DSP-201 (desmoplakin I) transcript, whereas most of exon 23 is excluded from the DSP-202 (desmoplakin II) transcript through alternative splicing. (**C**): Expanded schematic of *DSP* exon 23 showing the alternative splice donor site and the location of the c.4297C>T (p.Gln1433*) variant. The variant lies within the exon 23 region retained in DSP-201 but largely spliced out in DSP-202, consistent with an isoform-specific effect of the variant.

Figure 7 was designed to illustrate the transcript-specific impact of the *DSP* c.4297C>T (p.Gln1433*) variant. The *DSP* gene (ENSG00000120217), located on chromosome 6p24.3, consists of 24 exons (blue boxes), as illustrated in the University of California Santa Cruz Genome Browser. Exon 23 is highlighted to indicate the location of the identified variant c.4297C>T (p.Gln1433*; rs1554108283), which is marked by a red arrow. The corresponding protein domains are shown below, including the N-terminal head, central coiled-coil rod domain, linker region, and C-terminal domain (Figure 7A).

The genomic location of the *DSP* gene on chromosome 6 is illustrated in Figure 7A. The major *DSP* transcript variants demonstrate differential exon usage. Transcript variant 1 (*DSP*-201), encoding desmoplakin I (DSPI; 2871 amino acids), includes the full length of exon 23. The identified nonsense variant (p.Gln1433*) lies within this region (red box and star), resulting in a premature termination codon. In contrast, transcript variant 2 (*DSP*-202), encoding desmoplakin II (DSPII; 2272 amino acids), undergoes alternative splicing that removes most of exon 23 via an alternative donor site, thereby excluding the region harbouring the variant (Figure 7B).

The predicted protein consequences differ between transcripts. In DSPI, the c.4297C>T variant is expected to result in either nonsense-mediated mRNA decay or production of a truncated protein lacking essential C-terminal domains. In contrast, DSPII is predicted to remain unaffected, as the mutation lies within a region that is spliced out in this transcript (Figure 7C).

Finally, all available family members were screened to assess carrier status, including the parents (KU-48.III-1 and KU-48.III-2) and six unaffected siblings (KU-48.IV-2, IV-3, IV-5, IV-6, IV-7, and IV-10), as seen in Supplementary Figure S3. Five siblings were identified as heterozygous carriers, while one sibling, KU-48.IV-3, carried two copies of the wild-type allele. The high carrier frequency among siblings is consistent with autosomal recessive inheritance in a consanguineous pedigree.

To assess the potential clinical impact of heterozygosity, ECG and echocardiographic evaluations were performed in two carriers (the father, KU-48.III-1, and the unaffected brother, KU-48.IV-7); the results are shown in Supplementary Figures S4 and S5.

### 3.3. In Silico Assessment and ACMG/AMP Classification of the DSP c.4297C>T (p.Gln1433*) Variant

The identified *DSP* c.4297C>T (p.Gln1433)* variant introduces a premature termination codon within exon 23 of *DSP*. As exon 23 is included in the desmoplakin I (DSPI) transcript but excluded from the desmoplakin II (DSPII) transcript through alternative splicing, the variant is predicted to selectively disrupt DSPI while leaving the coding sequence of DSPII unaffected. The premature stop codon is expected to result in nonsense-mediated mRNA decay or production of a truncated protein lacking the C-terminal intermediate filament-binding domain, consistent with the established loss-of-function disease mechanism of DSP.

According to the ACMG/AMP guidelines, the variant was classified as pathogenic based on the combination of PVS1 (very strong), PM2 (moderate), PP1 (supporting), and PP4 (supporting) classifications. PVS1 was applied because the variant is a canonical nonsense variant predicted to result in loss of function, an established mechanism of disease for DSP. PM2 was assigned because the variant is absent or extremely rare in population databases. PP1 was supported by segregation within the investigated family, as all affected siblings are homozygous for the variant and both parents are heterozygous carriers. PP4 was applied because the affected individuals exhibited a phenotype highly specific for DSP-associated Carvajal syndrome, including arrhythmogenic cardiomyopathy, woolly hair, palmoplantar keratoderma, and characteristic histopathological findings. The *DSP* c.4297C>T (p.Gln1433*) was classified according to the ACMG/AMP guidelines, with the applied evidence criteria summarized in Table 3.

Computational annotation using multiple prediction algorithms uniformly supported a deleterious effect of the variant. However, because this is a canonical loss-of-function nonsense variant, these computational predictions provide supportive rather than primary evidence for pathogenicity. Detailed computational annotations are provided in the Supplementary Material and Supplementary Table S1.

## 4. Discussion

Our findings provide compelling evidence for a founder effect underlying Carvajal syndrome in this consanguineous Arabian family, with the homozygous pathogenic *DSP* variant localized within a shared ancestral chromosomal segment identified using autozygosity mapping. It is a powerful approach for identifying autosomal recessive disease loci in consanguineous families, where pathogenic variants are often located within ancestral homozygous chromosomal segments that are identical by descent (IBD) [36]. It is particularly useful in multiplex families with more than one affected individual. In this study, autozygosity mapping was performed using exome sequencing data. Although its resolution is lower than SNP-based linkage due to a higher proportion of “no-calls,” it was nevertheless effective in identifying the causative variant in this family. This approach has previously been successfully applied in consanguineous populations in Kuwait to map recessive pathogenic variants underlying congenital disorders [36,47,48].

Using this strategy, we identified a shared IBD interval spanning the *DSP* locus on chromosome 6 and subsequently detected a novel homozygous nonsense variant (c.4297C>T; p.Gln1433*; rs1554108283) in exon 23 of *DSP* in two affected siblings with arrhythmogenic cardiomyopathy (ACM). This variant is particularly relevant as it affects the DSPI isoform (transcript variant 1), the predominant cardiac isoform accounting for approximately 77% of *DSP* transcripts [49]. The resulting truncated transcript is unstable, leading to the loss of DSPI protein, while residual DSPII expression likely explains survival and the absence of fetal lethality [50]. The *DSP*:c.4297C>T (p.Gln1433*) variant introduces a premature termination codon within exon 23, a region that is included in the long desmoplakin I (DSPI) transcript but excluded from the shorter desmoplakin II (DSPII) transcript through physiological alternative splicing. Consequently, the variant is predicted to selectively disrupt DSPI while leaving the coding sequence of DSPII unaffected. As loss of function is an established disease mechanism for DSP-associated cardiomyopathy and Carvajal syndrome, this transcript-specific truncating variant provides a biologically plausible explanation for the severe cardiocutaneous phenotype observed in this family.

Importantly, the proposed mechanism does not involve aberrant RNA splicing. Instead, the transcript-specific effect arises from the normal alternative splicing of *DSP*, whereby exon 23 is incorporated into DSPI but excluded from DSPII. Therefore, the c.4297C>T variant only disrupts the coding sequence of DSPI without affecting canonical splice donor or acceptor sites. This interpretation is consistent with published RNA sequencing analyses demonstrating the physiological expression of both DSP isoforms in human myocardium, with DSPI representing the predominant transcript [49].

The molecular mechanism proposed here is supported by multiple independent lines of evidence, including segregation of the variant within the family, the highly characteristic phenotype of Carvajal syndrome, histopathological findings, and selective loss of desmoplakin I demonstrated in the present study. Collectively, these findings support the pathogenic role of the variant and are consistent with its classification as pathogenic according to ACMG/AMP criteria.

Although direct functional studies were not feasible in the present family, our observations are consistent with recent experimental studies using gene-edited induced pluripotent stem cell-derived cardiomyocytes carrying DSPI-specific truncating variants, which demonstrated selective disruption of desmoplakin I and associated cellular abnormalities. These experimental data provide independent support for the proposed pathogenic mechanism and strengthen the biological interpretation of our clinical observations.

DSP cardiomyopathy is typically characterized by subepicardial late gadolinium enhancement (LGE) on cardiac MRI, frequent premature ventricular contractions (PVCs), and a high risk of ventricular arrhythmias [23,31,32,33]. A female predominance has also been reported in several cohorts [31,33]. Most cases of Carvajal syndrome are caused by biallelic truncating variants in *DSP*, commonly located in exon 24, where premature stop codons are not subject to nonsense-mediated decay and instead produce C-terminally truncated proteins with residual function [51]. In contrast, the present case involves a DSPI-specific truncating variant, highlighting phenotypic diversity within DSP-related disease mechanisms [52].

The *DSP* gene encodes desmoplakin, a key desmosomal protein linking intermediate filaments to desmosomal plaques. Pathogenic variants are associated with cardiomyopathy, palmoplantar keratoderma (PPK), and woolly or curly hair [16,53]. Cutaneous manifestations are common in heterozygous carriers, with up to 44–55% showing PPK and/or hair abnormalities [31,32,33]. In this family, heterozygous carriers showed variable expressivity: one individual (KU-48.IV-7) had normal cardiac evaluation by ECG and echocardiography, while another (KU-48.III-1) developed later-onset, milder cardiomyopathy compared with the severe early-onset disease observed in homozygous siblings, demonstrating incomplete penetrance and gene dosage effects.

Inflammation-related myocardial injury is a recognized feature of DSP cardiomyopathy. Histological studies have demonstrated inflammatory infiltrates in affected myocardium, and clinical “hot phases” characterized by chest pain, troponin elevation, and ECG changes are being increasingly reported [21,29,54]. These episodes may mimic acute myocarditis and are associated with subepicardial LGE on CMR [12]. *DSP* variants are frequently enriched in patients presenting with such inflammatory episodes, accounting for a substantial proportion of cases in ACM and myocarditis cohorts [21,55]. Myocardial injury has been reported in 14–22% of *DSP* carriers and is strongly associated with ventricular arrhythmias, heart failure, and progressive fibrosis [23,31,32,33,49]. These findings highlight the importance of genetic testing in patients with recurrent or atypical myocarditis, particularly in familial cases [56,57,58].

Electrocardiographic abnormalities in DSP cardiomyopathy reflect progressive fibrofatty myocardial replacement. The most common findings include low QRS voltages and T-wave inversions in precordial and inferior leads, which are present in a substantial proportion of patients [21,32].

In pediatric patients, T-wave inversions may represent normal age-related variants. PR intervals are typically within the normal range, although mild prolongation can occur, as was observed in KU.48.IV-9. Epsilon waves are uncommon and are usually associated with right ventricular involvement. Importantly, ECG abnormalities may precede overt structural disease, particularly in heterozygous carriers. In contrast, homozygous or biallelic cases tend to present earlier and with a more aggressive phenotype due to more pronounced desmoplakin deficiency, whereas heterozygous individuals generally exhibit later onset and milder disease.

The mildly prolonged PR interval in this case is most consistent with an intrinsic feature of DSP-related cardiomyopathy rather than a treatment effect, as the patient was not receiving any negative dromotropic medications, including amiodarone, at the time of ECG acquisition. Furthermore, the upper limit of normal for the patient’s age is approximately 190 ms, supporting the interpretation of a true conduction delay [59].

Ventricular arrhythmias are a major clinical feature of DSP cardiomyopathy. Heterozygous carriers have a high incidence of premature ventricular contractions and sustained ventricular tachyarrhythmias, including ventricular tachycardia and ventricular fibrillation [18,21,27]. Reported rates of malignant arrhythmias range from 29% to 46%, often occurring at a young age and sometimes in the absence of significant left ventricular dysfunction [32]. In the present family, no ventricular arrhythmias were documented in heterozygous carriers during the available follow-up.

Cardiac magnetic resonance (CMR) is the most sensitive imaging modality for DSP cardiomyopathy. A characteristic feature is subepicardial LGE, often involving multiple left ventricular segments, with a predilection for the inferolateral and inferoseptal walls [23,31,32,33]. In early disease, left ventricular size and systolic function may remain normal despite the presence of extensive fibrosis, explaining the limited sensitivity of echocardiography [3,26,31]. LGE is reported in up to 40–72% of patients and may follow a circumferential “ring-like” pattern, particularly in DSP and FLNC-related disease [3,24,31,32,33]. Importantly, the extent of fibrosis often exceeds the degree of systolic dysfunction and is strongly associated with arrhythmic risk. Histopathological studies confirm midmyocardial and subepicardial fibrofatty replacement as the dominant pattern of disease [24,60].

In conclusion, this study highlights the value of integrating autozygosity mapping with exome sequencing to efficiently identify disease-causing variants in consanguineous families. This combined approach led to the discovery of a novel homozygous DSPI-specific truncating variant in *DSP*, expanding the known mutational and phenotypic spectrum of desmoplakin cardiomyopathy. The findings illustrate marked intrafamilial variability, ranging from severe early-onset disease in homozygous individuals to milder or subclinical manifestations in heterozygous carriers, and reinforce the importance of *DSP* in arrhythmogenic and myocarditis-like phenotypes. Overall, this work supports the clinical utility of genomic analysis in unexplained cardiomyopathy and recurrent myocarditis, particularly in genetically enriched populations, and contributes to improved molecular diagnosis and an improved understanding of disease mechanisms.

## Figures and Tables

**Table 1:** Summary of clinical, electrocardiographic, and imaging findings in the affected siblings (KU-48.IV-8 and KU-48.IV-9).

Feature	KU-48.IV-8	KU-48.IV-9
Age of clinical onset	6 years	7 years
Age at death	11 years	12 years
Clinical presentation	Recurrent chest pain, shortness of breath, congestive heart failure	Recurrent chest pain, shortness of breath, congestive heart failure, ventricular tachycardia
Baseline ECG	Incomplete right bundle branch block; premature ventricular complexes	Epsilon wave; premature ventricular complexes
Echocardiography	Dilated left ventricle with systolic dysfunction; right ventricular trabeculations	Dilated left ventricle with systolic dysfunction; apical thrombus; right ventricular dilatation and systolic dysfunction
Cardiac MRI	Not available	Dilated right ventricle; RVEF of 25%; extensive late gadolinium enhancement involving the entire left ventricular lateral wall and right ventricular apex

**Table 2:** A summary of the shared autosomal identity-by-descent (IBD) intervals in the affected siblings, showing chromosome number, genomic start and end positions, and interval size in megabases (Mb).

Chr No.	Start Position (bp)	End Position (bp)	Interval (Mb)
2	195,926,167	206,157,529	10.23
3	7,459,566	12,173,124	4.71
6	2,774,723	10,721,339	7.95
7	117,778,787	129,464,259	11.69
16	1,745,032	5,919,853	4.17
16	46,349,053	77,141,857	30.79

**Table 3:** ACMG/AMP classification of the *DSP*:c.4297C>T (p.Gln1433*) variant based on the applied evidence criteria supporting its pathogenic classification.

ACMG Criterion	Strength	Evidence
PVS1	Very Strong	Canonical nonsense variant predicted to cause loss of function through premature termination codon/NMD. Loss of function is an established disease mechanism for DSP.
PM2	Moderate	Absent or extremely rare in population databases.
PP1	Supporting	Segregates with disease within the family (affected siblings homozygous; parents heterozygous).
PP4	Supporting	Highly specific phenotype consistent with Carvajal syndrome caused by *DSP* variants.
Classification	Pathogenic	PVS1 + PM2 + PP1 + PP4

## Data Availability

Raw sequencing data involves identifiable human genetic information which cannot be publicly released.

## Supplementary Materials

The following supporting information can be downloaded at: https://www.mdpi.com/article/10.3390/jcm15155933/s1 [39,40,41,42,43,44,45], Figure S1: ECG findings in affected siblings; Figure S2: Shared identity-by-descent (IBD) intervals identified across autosomal chromosomes in the two affected siblings; Figure S3: *DSP* variants chromatographs for the six non-affected siblings of Family KU-48; Figure S4: Electrocardiogram and Echocardiograms for the non-affected father KU-48.III-1; Figure S5: Electrocardiogram and Echocardiograms for the non-affected male sibling KU-48.IV-7; Table S1: Summarises the in silico analyses for *DSP*:c.4297C>T, showing the prediction tools that have the most significant pathogenicity scores; Supplementary Video S1: Cardiac magnetic resonance cine imaging of patient KU-48.IV-9 demonstrating severe right ventricular systolic dysfunction with regional wall motion abnormalities consistent with arrhythmogenic cardiomyopathy.

## References

[1] Gross A., Zhou B., Bewersdorf L., Schwarz N., Schacht G.M., Boor P., Hoeft K., Hoffmann B., Fuchs E., Kramann R. (2022). Desmoplakin Maintains Transcellular Keratin Scaffolding and Protects From Intestinal Injury. Cell. Mol. Gastroenterol. Hepatol..

[2] Bariani R., Rigato I., Cason M., Marinas M.B., Celeghin R., Pilichou K., Bauce B. (2022). Genetic Background and Clinical Features in Arrhythmogenic Left Ventricular Cardiomyopathy: A Systematic Review. J. Clin. Med..

[3] Brandao M., Bariani R., Rigato I., Bauce B. (2023). Desmoplakin Cardiomyopathy: Comprehensive Review of an Increasingly Recognized Entity. J. Clin. Med..

[4] Ebrahim M.A., Ali N.M., Albash B.Y., Al Sayegh A.H., Ahmad N.B., Voss S., Klag F., Gross J., Holler S., Walhorn V. (2025). Phenotypic Diversity Caused by the DES Missense Mutation p.R127P (c.380G>C) Contributing to Significant Cardiac Mortality and Morbidity Associated With a Desmin Filament Assembly Defect. Circ. Genom. Precis. Med..

[5] Bermudez-Jimenez F.J., Carriel V., Brodehl A., Alaminos M., Campos A., Schirmer I., Milting H., Abril B.A., Alvarez M., Lopez-Fernandez S. (2018). Novel Desmin Mutation p.Glu401Asp Impairs Filament Formation, Disrupts Cell Membrane Integrity, and Causes Severe Arrhythmogenic Left Ventricular Cardiomyopathy/Dysplasia. Circulation.

[6] Nekrasova O., Harmon R.M., Broussard J.A., Koetsier J.L., Godsel L.M., Fitz G.N., Gardel M.L., Green K.J. (2018). Desmosomal cadherin association with Tctex-1 and cortactin-Arp2/3 drives perijunctional actin polymerization to promote keratinocyte delamination. Nat. Commun..

[7] Clemen C.S., Herrmann H., Strelkov S.V., Schroder R. (2013). Desminopathies: Pathology and mechanisms. Acta Neuropathol..

[8] Gerull B., Brodehl A. (2021). Insights Into Genetics and Pathophysiology of Arrhythmogenic Cardiomyopathy. Curr. Heart Fail. Rep..

[9] Cipriani A., Mattesi G., Bariani R., Cecere A., Martini N., De Michieli L., Da Pozzo S., Corradin S., De Conti G., Zorzi A. (2023). Cardiac magnetic resonance imaging of arrhythmogenic cardiomyopathy: Evolving diagnostic perspectives. Eur. Radiol..

[10] Cabral R.M., Wan H., Cole C.L., Abrams D.J., Kelsell D.P., South A.P. (2010). Identification and characterization of DSPIa, a novel isoform of human desmoplakin. Cell Tissue Res..

[11] Yuan Z.Y., Cheng L.T., Wang Z.F., Wu Y.Q. (2021). Desmoplakin and clinical manifestations of desmoplakin cardiomyopathy. Chin. Med. J. (Engl.).

[12] Norgett E.E., Hatsell S.J., Carvajal-Huerta L., Cabezas J.C., Common J., Purkis P.E., Whittock N., Leigh I.M., Stevens H.P., Kelsell D.P. (2000). Recessive mutation in desmoplakin disrupts desmoplakin-intermediate filament interactions and causes dilated cardiomyopathy, woolly hair and keratoderma. Hum. Mol. Genet..

[13] Yermakovich D., Sivitskaya L., Vaikhanskaya T., Danilenko N., Motuk I. (2018). Novel desmoplakin mutations in familial Carvajal syndrome. Acta Myol..

[14] Favre B., Begre N., Borradori L. (2018). A recessive mutation in the DSP gene linked to cardiomyopathy, skin fragility and hair defects impairs the binding of desmoplakin to epidermal keratins and the muscle-specific intermediate filament desmin. Br. J. Dermatol..

[15] Ramoglu M.G., Ucar T., Ceylaner S., Atalay S., Tutar E. (2017). A novel mutation in the desmoplakin gene in two female siblings with a rare form of dilated cardiomyopathy: Carvajal syndrome. Anatol. J. Cardiol..

[16] Pigors M., Schwieger-Briel A., Cosgarea R., Diaconeasa A., Bruckner-Tuderman L., Fleck T., Has C. (2015). Desmoplakin mutations with palmoplantar keratoderma, woolly hair and cardiomyopathy. Acta Derm. Venereol..

[17] McKoy G., Protonotarios N., Crosby A., Tsatsopoulou A., Anastasakis A., Coonar A., Norman M., Baboonian C., Jeffery S., McKenna W.J. (2000). Identification of a deletion in plakoglobin in arrhythmogenic right ventricular cardiomyopathy with palmoplantar keratoderma and woolly hair (Naxos disease). Lancet.

[18] Corrado D., Perazzolo Marra M., Zorzi A., Beffagna G., Cipriani A., Lazzari M., Migliore F., Pilichou K., Rampazzo A., Rigato I. (2020). Diagnosis of arrhythmogenic cardiomyopathy: The Padua criteria. Int. J. Cardiol..

[19] Marcus F.I., McKenna W.J., Sherrill D., Basso C., Bauce B., Bluemke D.A., Calkins H., Corrado D., Cox M.G., Daubert J.P. (2010). Diagnosis of arrhythmogenic right ventricular cardiomyopathy/dysplasia: Proposed modification of the task force criteria. Circulation.

[20] Rampazzo A., Nava A., Malacrida S., Beffagna G., Bauce B., Rossi V., Zimbello R., Simionati B., Basso C., Thiene G. (2002). Mutation in human desmoplakin domain binding to plakoglobin causes a dominant form of arrhythmogenic right ventricular cardiomyopathy. Am. J. Hum. Genet..

[21] Bauce B., Basso C., Rampazzo A., Beffagna G., Daliento L., Frigo G., Malacrida S., Settimo L., Danieli G., Thiene G. (2005). Clinical profile of four families with arrhythmogenic right ventricular cardiomyopathy caused by dominant desmoplakin mutations. Eur. Heart J..

[22] Sen-Chowdhry S., Syrris P., Ward D., Asimaki A., Sevdalis E., McKenna W.J. (2007). Clinical and genetic characterization of families with arrhythmogenic right ventricular dysplasia/cardiomyopathy provides novel insights into patterns of disease expression. Circulation.

[23] Lopez-Ayala J.M., Gomez-Milanes I., Sanchez Munoz J.J., Ruiz-Espejo F., Ortiz M., Gonzalez-Carrillo J., Lopez-Cuenca D., Oliva-Sandoval M.J., Monserrat L., Valdes M. (2014). Desmoplakin truncations and arrhythmogenic left ventricular cardiomyopathy: Characterizing a phenotype. Europace.

[24] Augusto J.B., Eiros R., Nakou E., Moura-Ferreira S., Treibel T.A., Captur G., Akhtar M.M., Protonotarios A., Gossios T.D., Savvatis K. (2020). Dilated cardiomyopathy and arrhythmogenic left ventricular cardiomyopathy: A comprehensive genotype-imaging phenotype study. Eur. Heart J. Cardiovasc. Imaging.

[25] Pinto Y.M., Elliott P.M., Arbustini E., Adler Y., Anastasakis A., Bohm M., Duboc D., Gimeno J., de Groote P., Imazio M. (2016). Proposal for a revised definition of dilated cardiomyopathy, hypokinetic non-dilated cardiomyopathy, and its implications for clinical practice: A position statement of the ESC working group on myocardial and pericardial diseases. Eur. Heart J..

[26] Cipriani A., Bauce B., De Lazzari M., Rigato I., Bariani R., Meneghin S., Pilichou K., Motta R., Aliberti C., Thiene G. (2020). Arrhythmogenic Right Ventricular Cardiomyopathy: Characterization of Left Ventricular Phenotype and Differential Diagnosis With Dilated Cardiomyopathy. J. Am. Heart Assoc..

[27] Corrado D., Basso C. (2022). Arrhythmogenic left ventricular cardiomyopathy. Heart.

[28] Protonotarios N., Tsatsopoulou A. (2004). Naxos disease and Carvajal syndrome: Cardiocutaneous disorders that highlight the pathogenesis and broaden the spectrum of arrhythmogenic right ventricular cardiomyopathy. Cardiovasc. Pathol..

[29] Castelletti S., Vischer A.S., Syrris P., Crotti L., Spazzolini C., Ghidoni A., Parati G., Jenkins S., Kotta M.C., McKenna W.J. (2017). Desmoplakin missense and non-missense mutations in arrhythmogenic right ventricular cardiomyopathy: Genotype-phenotype correlation. Int. J. Cardiol..

[30] Gigli M., Merlo M., Graw S.L., Barbati G., Rowland T.J., Slavov D.B., Stolfo D., Haywood M.E., Dal Ferro M., Altinier A. (2019). Genetic Risk of Arrhythmic Phenotypes in Patients With Dilated Cardiomyopathy. J. Am. Coll. Cardiol..

[31] Smith E.D., Lakdawala N.K., Papoutsidakis N., Aubert G., Mazzanti A., McCanta A.C., Agarwal P.P., Arscott P., Dellefave-Castillo L.M., Vorovich E.E. (2020). Desmoplakin Cardiomyopathy, a Fibrotic and Inflammatory Form of Cardiomyopathy Distinct From Typical Dilated or Arrhythmogenic Right Ventricular Cardiomyopathy. Circulation.

[32] Bariani R., Cason M., Rigato I., Cipriani A., Celeghin R., De Gaspari M., Bueno Marinas M., Mattesi G., Pergola V., Rizzo S. (2022). Clinical profile and long-term follow-up of a cohort of patients with desmoplakin cardiomyopathy. Heart Rhythm..

[33] Wang W., Murray B., Tichnell C., Gilotra N.A., Zimmerman S.L., Gasperetti A., Scheel P., Tandri H., Calkins H., James C.A. (2022). Clinical characteristics and risk stratification of desmoplakin cardiomyopathy. Europace.

[34] Carr I.M., Morgan J., Watson C., Melnik S., Diggle C.P., Logan C.V., Harrison S.M., Taylor G.R., Pena S.D., Markham A.F. (2013). Simple and efficient identification of rare recessive pathologically important sequence variants from next generation exome sequence data. Hum. Mutat..

[35] Watson C.M., Crinnion L.A., Gurgel-Gianetti J., Harrison S.M., Daly C., Antanavicuite A., Lascelles C., Markham A.F., Pena S.D., Bonthron D.T. (2015). Rapid Detection of Rare Deleterious Variants by Next Generation Sequencing with Optional Microarray SNP Genotype Data. Hum. Mutat..

[36] Al-Mutairi D.A., Alsabah B.H., Alkhaledi B.A., Pennekamp P., Omran H. (2022). Identification of a novel founder variant in DNAI2 cause primary ciliary dyskinesia in five consanguineous families derived from a single tribe descendant of Arabian Peninsula. Front. Genet..

[37] Carr I.M., Camm N., Taylor G.R., Charlton R., Ellard S., Sheridan E.G., Markham A.F., Bonthron D.T. (2011). GeneScreen: A program for high-throughput mutation detection in DNA sequence electropherograms. J. Med. Genet..

[38] Rotunda A.M., Craft N., Haley J.C. (2004). Hyperkeratotic plaques on the palms and soles. Palmoplantar lichen planus, hyperkeratotic variant. Arch. Dermatol..

[39] Schwarz J.M., Cooper D.N., Schuelke M., Seelow D. (2014). MutationTaster2: Mutation prediction for the deep-sequencing age. Nat. Methods.

[40] Kircher M., Witten D.M., Jain P., O’Roak B.J., Cooper G.M., Shendure J. (2014). A general framework for estimating the relative pathogenicity of human genetic variants. Nat. Genet..

[41] Shihab H.A., Gough J., Mort M., Cooper D.N., Day I.N., Gaunt T.R. (2014). Ranking non-synonymous single nucleotide polymorphisms based on disease concepts. Hum. Genom..

[42] Ionita-Laza I., McCallum K., Xu B., Buxbaum J.D. (2016). A spectral approach integrating functional genomic annotations for coding and noncoding variants. Nat. Genet..

[43] Quang D., Chen Y., Xie X. (2015). DANN: A deep learning approach for annotating the pathogenicity of genetic variants. Bioinformatics.

[44] Gulko B., Hubisz M.J., Gronau I., Siepel A. (2015). A method for calculating probabilities of fitness consequences for point mutations across the human genome. Nat. Genet..

[45] Chun S., Fay J.C. (2009). Identification of deleterious mutations within three human genomes. Genome Res..

[46] Carr I.M., Bhaskar S., James O.S., Aldahmesh M.A., Shamseldin H.E., Markham A.F., Bonthron D.T., Black G., Alkuraya F.S. (2012). Autozygosity Mapping with Exome Sequence Data. Hum. Mutat..

[47] Wallmeier J., Al-Mutairi D.A., Chen C.T., Loges N.T., Pennekamp P., Menchen T., Ma L., Shamseldin H.E., Olbrich H., Dougherty G.W. (2014). Mutations in CCNO result in congenital mucociliary clearance disorder with reduced generation of multiple motile cilia. Nat. Genet..

[48] Al-Mutairi D.A., Alsabah B.H., Pennekamp P., Omran H. (2023). Mapping the Most Common Founder Variant in RSPH9 That Causes Primary Ciliary Dyskinesia in Multiple Consanguineous Families of Bedouin Arabs. J. Clin. Med..

[49] Smith E.D., Jin K., Ferguson B., Tsan Y.C., DePalma S.J., Meisner J., Renberg A., Bedi K., Friedline S., Margulies K.B. (2025). Desmoplakin Haploinsufficiency Underlies Cell-Cell Adhesion Failure in DSP Cardiomyopathy and is Rescued by Transcriptional Activation. bioRxiv.

[50] Uzumcu A., Norgett E.E., Dindar A., Uyguner O., Nisli K., Kayserili H., Sahin S.E., Dupont E., Severs N.J., Leigh I.M. (2006). Loss of desmoplakin isoform I causes early onset cardiomyopathy and heart failure in a Naxos-like syndrome. J. Med. Genet..

[51] Pantou M.P., Gourzi P., Vlagkouli V., Papatheodorou E., Tsoutsinos A., Nyktari E., Degiannis D., Anastasakis A. (2023). A truncating variant altering the extreme C-terminal region of desmoplakin (DSP) suggests the crucial functional role of the region: A case report study. BMC Med. Genom..

[52] Arici S., Akalin F., Geckinli B.B. (2024). A case of Carvajal syndrome presenting with dilated cardiomyopathy. Cardiol. Young.

[53] Yao J.V., Winship I. (2020). More than meets the eye: Palmoplantar keratoderma and arrhythmogenic right ventricular cardiomyopathy in a patient with loss of the DSP gene. JAAD Case Rep..

[54] Reza N., de Feria A., Chowns J.L., Hoffman-Andrews L., Vann L., Kim J., Marzolf A., Owens A.T. (2022). Cardiovascular Characteristics of Patients with Genetic Variation in Desmoplakin (DSP). Cardiogenetics.

[55] Bariani R., Rigato I., Cipriani A., Bueno Marinas M., Celeghin R., Basso C., Corrado D., Pilichou K., Bauce B. (2022). Myocarditis-like Episodes in Patients with Arrhythmogenic Cardiomyopathy: A Systematic Review on the So-Called Hot-Phase of the Disease. Biomolecules.

[56] Bariani R., Cipriani A., Rizzo S., Celeghin R., Bueno Marinas M., Giorgi B., De Gaspari M., Rigato I., Leoni L., Zorzi A. (2021). ‘Hot phase’ clinical presentation in arrhythmogenic cardiomyopathy. Europace.

[57] Lota A.S., Hazebroek M.R., Theotokis P., Wassall R., Salmi S., Halliday B.P., Tayal U., Verdonschot J., Meena D., Owen R. (2022). Genetic Architecture of Acute Myocarditis and the Overlap With Inherited Cardiomyopathy. Circulation.

[58] Ammirati E., Raimondi F., Piriou N., Sardo Infirri L., Mohiddin S.A., Mazzanti A., Shenoy C., Cavallari U.A., Imazio M., Aquaro G.D. (2022). Acute Myocarditis Associated With Desmosomal Gene Variants. JACC Heart Fail..

[59] Saarel E.V., Granger S., Kaltman J.R., Minich L.L., Tristani-Firouzi M., Kim J.J., Ash K., Tsao S.S., Berul C.I., Stephenson E.A. (2018). Electrocardiograms in Healthy North American Children in the Digital Age. Circ. Arrhythm. Electrophysiol..

[60] Corrado D., Basso C., Thiene G., McKenna W.J., Davies M.J., Fontaliran F., Nava A., Silvestri F., Blomstrom-Lundqvist C., Wlodarska E.K. (1997). Spectrum of clinicopathologic manifestations of arrhythmogenic right ventricular cardiomyopathy/dysplasia: A multicenter study. J. Am. Coll. Cardiol..

