# Supplementary material for: Homozygous Nonsense DSP Variants Selectively Affecting Desmoplakin I Cause Carvajal Syndrome in a Consanguineous Arabian Family with Four Affected Female Siblings
Source: J Clin Med. 2026 Jul 29;15(15):5933. doi: 10.3390/jcm15155933 (PMC13466301; doi:10.3390/jcm15155933)
Supplement: Supplementary file 1 [file jcm-15-05933-s001.zip › Supplementary Figures.pdf]

Supplementary Online Information

Homozygous Nonsense *DSP* variants Selectively Affecting Desmoplakin I Cause Carvajal Syndrome in Consanguineous Arabian Family with Four Affected Female Siblings

Dalal A. Al-Mutairi [1,2,*], Mustafa A. Al-Qbandi [2], Ahmed AlTurki [3], Athbi A. Naief [4] and Adam S. Helms [5]

1 Human Genetics Unit, Faculty of Medicine, Kuwait University, Kuwait City 13110, Kuwait.
2 Department of Pediatrics Cardiology, Chest Diseases Hospital, Al-Sabah Medical district, Kuwait City 70001, Kuwait.
3 Department of Medicine, Faculty of Medicine, Kuwait University, Kuwait City 13110, Kuwait.
4 Department of Radiology, Al Sabah Hospital, Kuwait City 13041, Kuwait.
5 Department of Internal Medicine, Division of Cardiovascular Medicine, University of Michigan, Ann Arbor, USA.
* Correspondence: author: dalal.almutairi@ku.edu.kw

Supplementary figures

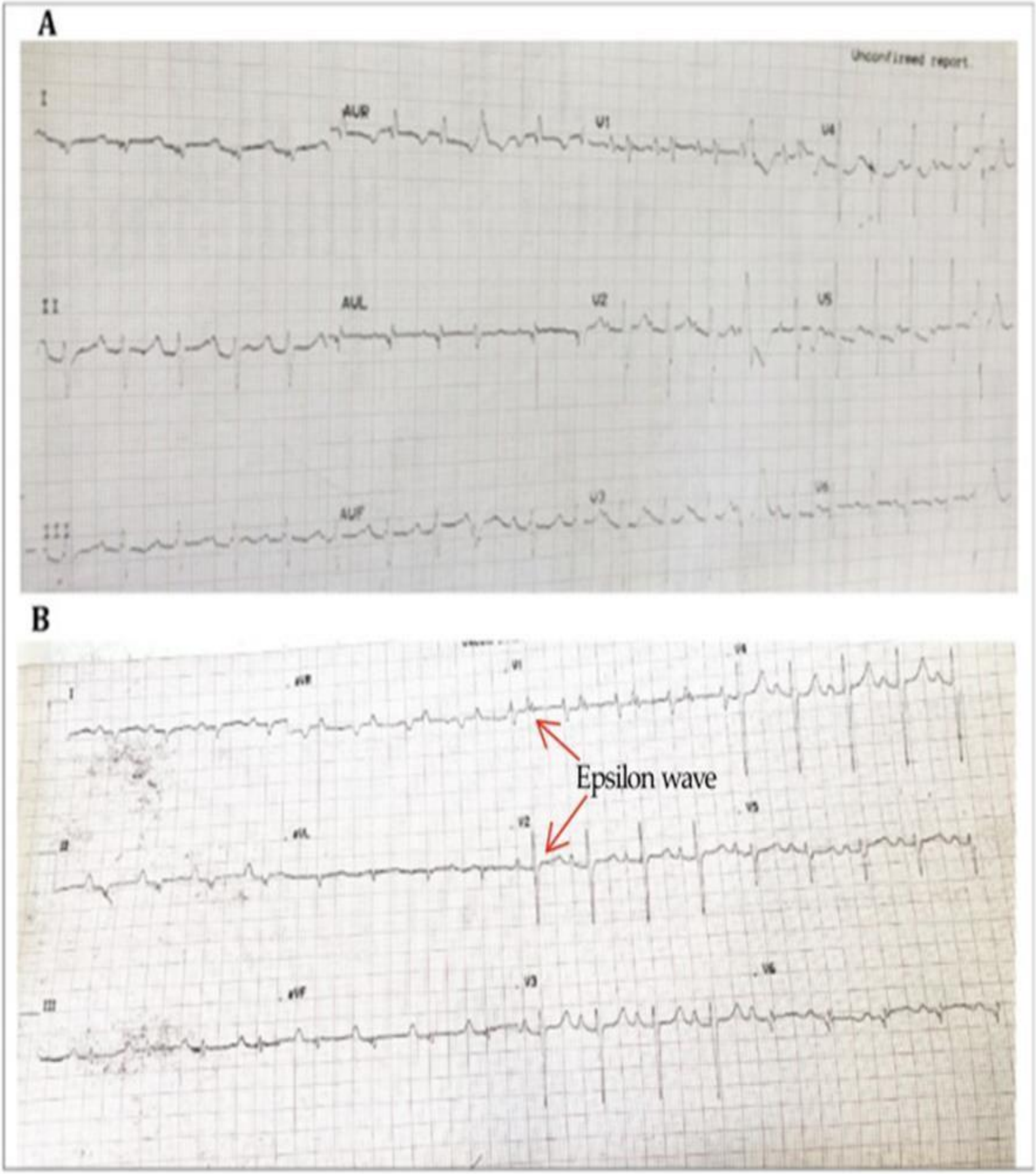

Figure S1. ECG findings in affected siblings: (A) KU-48.IV-8 shows an incomplete right bundle branch block, as well as premature ventricular complexes (PVCs) with a right ventricular morphology. (B) KU-48.IV-9 shows an epsilon wave.

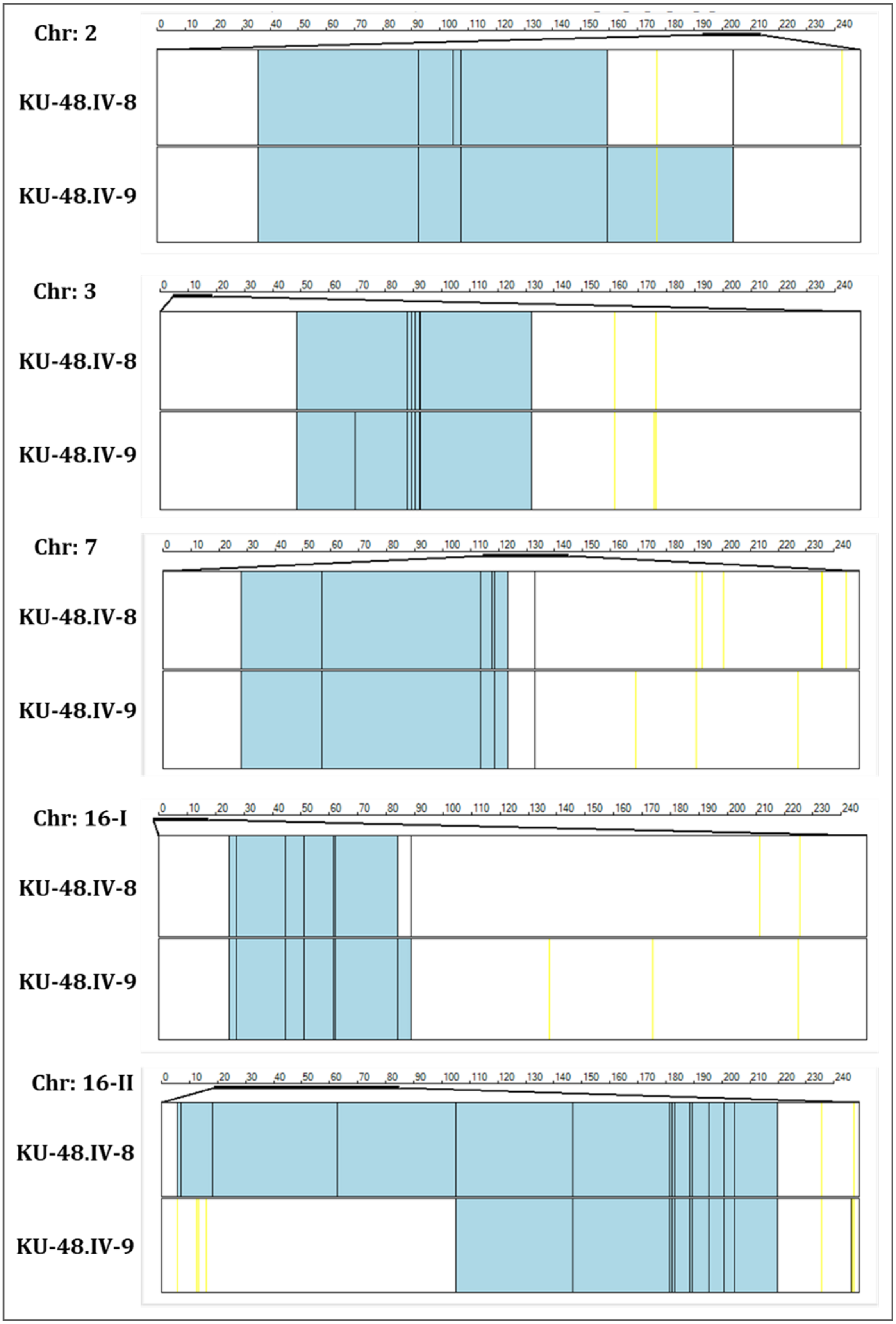

Figure S2. Shared identity-by-descent (IBD) intervals identified across autosomal chromosomes in the two affected siblings. Autozygosity mapping and linkage analysis revealed shared intervals on chromosomes 2, 3, 7, and 16.

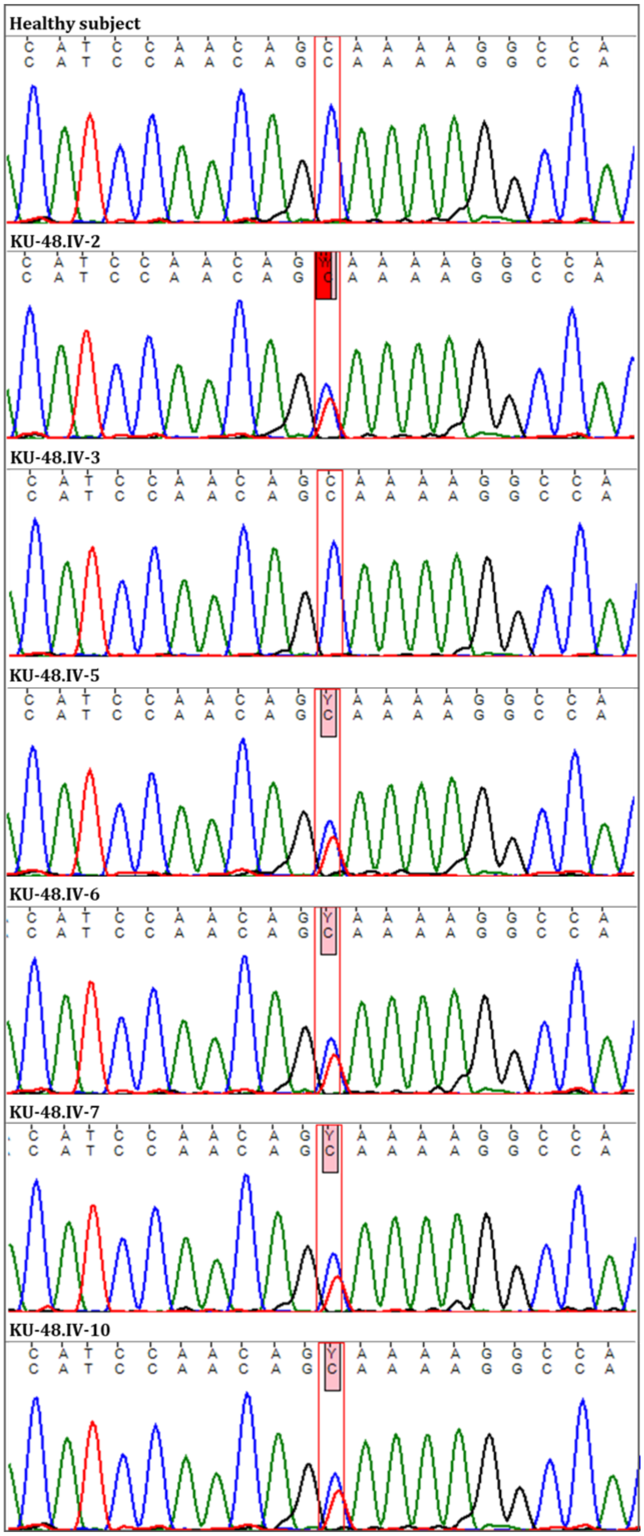

Figure S3. *DSP* variants chromatographs for the six non-affected siblings of Family KU-48.

It represents the chromatograms for the healthy subject and six of the non-affected siblings. Chromatograms show that five of the non-affected siblings (KU-48.IV-2, KU-48.IV-5, KU-48.IV-6, KU-48.IV-7and KU-48.IV-10) are heterozygous carriers for a founder nonsense variant (c.4297C>T; p.Gln1433*; rs1554108283) in exon 23 of *DSP* that results in predicts a premature termination of translation. The eldest sibling KU-48.IV-3 carries the two wild type alleles similarly to the healthy subject that also carries the two normal wild type alleles. At the pathogenic variant locus, pink rectangles indicate heterozygous variants, whereas red rectangles indicate homozygous pathogenic variants. The two red lines indicate the nucleotide position that differs from the wild-type allele.

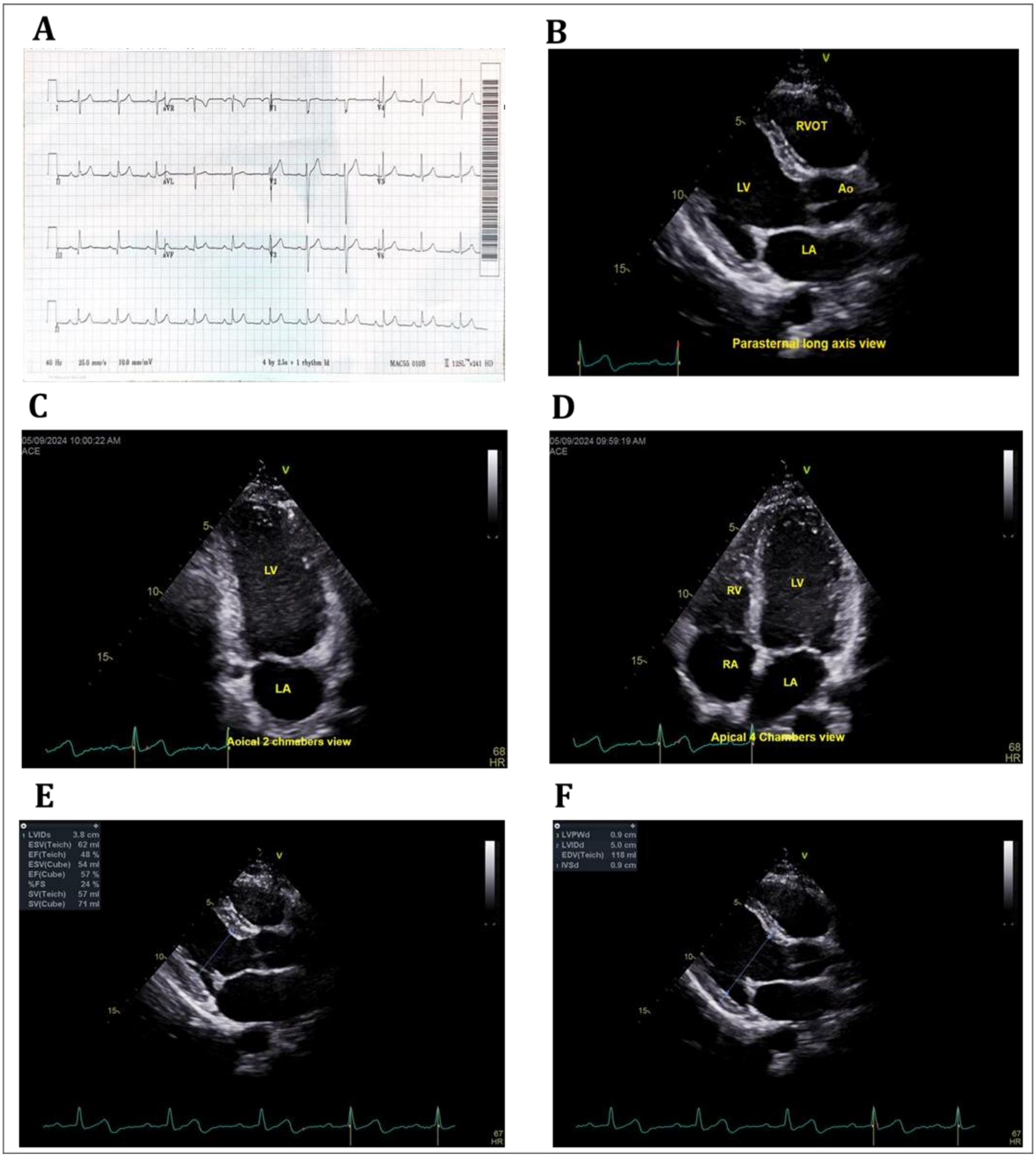

Figure S4. Electrocardiogram and Echocardiograms for the non-affected father KU-48.III-1.

Figure S4A) ECG findings show normal sinus rhythm, no abnormalities detected. Fig S4B) Echocardiogram for Apical 4 chamber view showing normal left ventricle (LV) size and wall thickness, mild impaired systolic function and mild global hypokinesia. EF 45-50%. Right ventricle (RV) normal in size with overall mildly impaired systolic function. Fig S4C) Echocardiogram for Apical 2 chamber view showing normal left ventricle size and wall thickness. mild impaired systolic function and mild global hypokinesia. EF 45-50%. Fig 12D) Echocardiogram show labelled parasternal long axis view. Fig S4 (E and F) Echocardiograms for parasternal long axis view at systole image (1) and diastole image (2) showing mild impaired systolic function and mild global hypokinesia. EF 45-50%. Remark: It was done at the age of 62.

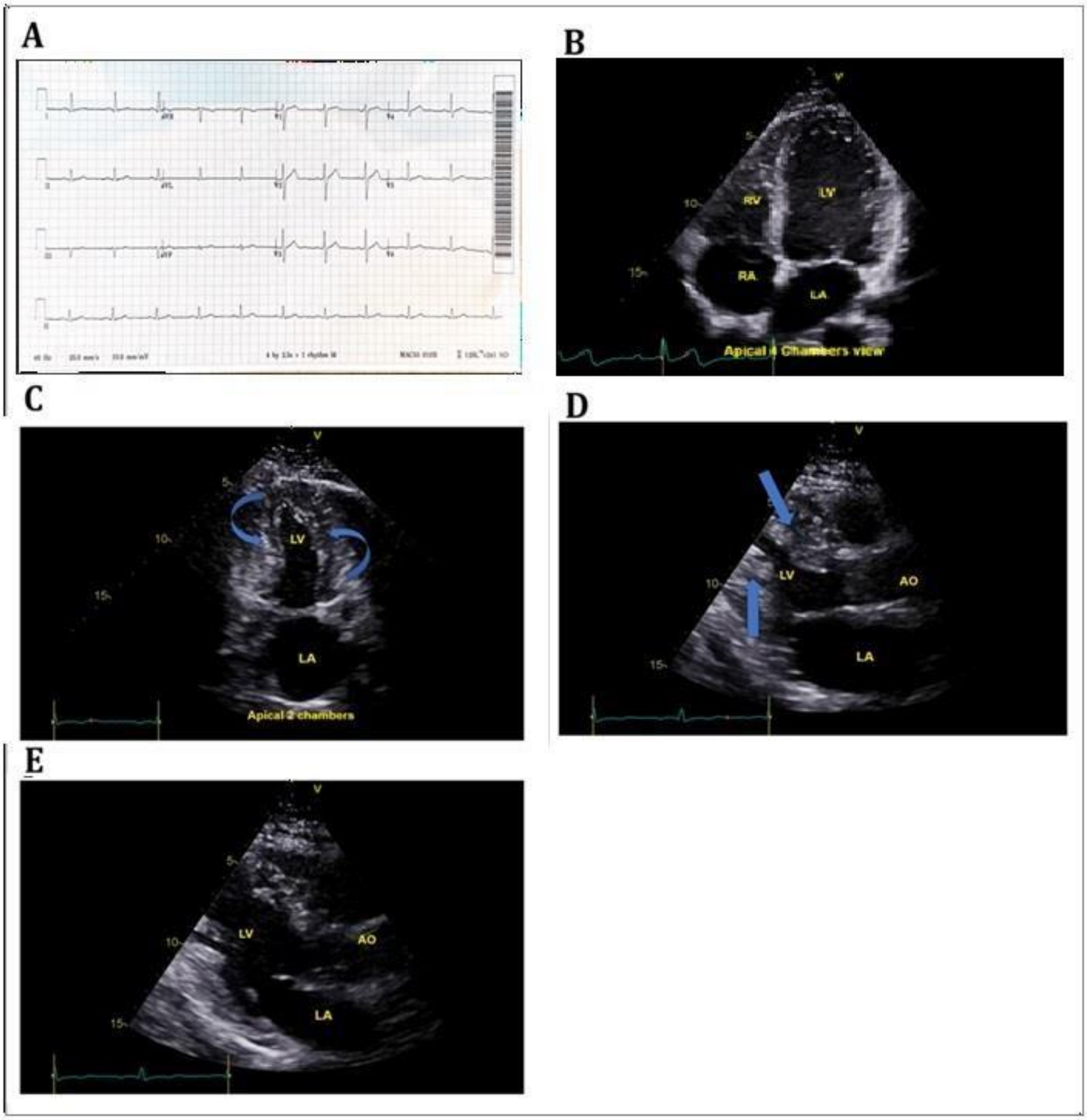

Figure S5. Electrocardiogram and Echocardiograms for the non-affected male sibling KU-48.IV-7.

Fig S5A) ECG findings show normal sinus rhythm, no abnormalities detected. Fig S5B) Echocardiogram for Apical 4 chamber view showing normal left ventricle (LV) size. There is borderline concentric left ventricular hypertrophy. Left atrium (LA) is mildly dilated. Left ventricular systolic function = 70%. Fig S5C) Echocardiogram for Apical 2 chamber view showing normal left ventricle (LV) size. There is borderline concentric left ventricular hypertrophy (arrow). Left atrium (LA) is mildly dilated. Fig S5 (D and E) Echocardiograms for Labelled parasternal long axis view. Parasternal long axis view at systole image (1) and diastole image (2) showing normal left ventricle (LV) size. There is borderline concentric left ventricular hypertrophy (arrow) . Left atrium (LA) is mildly dilated. Remark: It was done at the age of 19.

In silico pathogenicity prediction of the DSP variant

The in silico assessment of the *DSP* nonsense variant c.4297C>T (p.Gln1433*) in exon 23 was performed using multiple complementary pathogenicity prediction algorithms. The variant effect prediction included MutationTaster, which classifies variants as disease-causing or benign based on evolutionary conservation, splice-site alterations, protein features, and disease annotations [39]. Global deleteriousness was evaluated using the Combined Annotation Dependent Depletion (CADD) framework, which aggregates diverse genomic annotations into a single metric [40]. The functional impact was also assessed using FATHMM-MKL (coding), a machine learning-based classifier that incorporates sequence conservation and regulatory information [41]. Additional ensemble and unsupervised predictors included Eigen-PC, which applies principal component analysis to functional genomic annotations [42], and DANN, a deep neural network-based model trained to distinguish deleterious variants from neutral ones (rank score = 0.87558) [43]. The evolutionary constraint and functional importance of the affected genomic position were examined using the integrated fitCons score, which estimates the probability of fitness consequences based on functional genomic data [44]. Selective pressure at the nucleotide level was evaluated using the Likelihood Ratio Test (LRT), which compares neutral and constrained evolutionary models [45]. The complete data evidence assessment and applied criteria are provided in Supplementary Table S1.

MutationTaster: The *DSP* c.4297C>T (p.Gln1433*) variant is predicted to be disease-causing by MutationTaster, with a score of 1.0 indicating the highest level of confidence in pathogenicity. MutationTaster integrates information on evolutionary conservation, splice-site changes, protein truncation and known disease annotations. As a nonsense variant, p.Gln1433* introduces a premature termination codon in exon 23 of *DSP*. This is expected to result in either nonsense-mediated mRNA decay (NMD), or the production of a severely truncated desmoplakin protein that lacks essential C-terminal intermediate filament-binding domains. These loss-of-function (LoF) mechanisms are well established in *DSP*-related cardiocutaneous disorders, including Carvajal syndrome, thus supporting the MutationTaster prediction [39].

CADD (Combined Annotation Dependent Depletion): This variant has a high CADD raw rank score of 0.98556, which places it in the top 1–2% of the most deleterious variants in the human genome. CADD uses multiple functional, evolutionary and epigenetic annotations to estimate the likelihood that a variant is deleterious. Nonsense variants in genes with strong loss-of-function (LoF) disease mechanisms, such as *DSP*, typically achieve high CADD scores due to their substantial predicted impact on protein structure and gene function. The high score strongly supports the pathogenic nature of p.Gln1433*, which is consistent with the established correlations between genotype and phenotype in desmoplakin-related disorders [40].

FATHMM-MKL (coding): A FATHMM-MKL coding score of 0.99201 indicates a high probability of pathogenicity. FATHMM-MKL uses a machine learning framework that incorporates sequence conservation and regulatory features in order to distinguish between pathogenic and benign variants. The near-maximal score reflects the severe functional consequences of a premature stop codon within a highly constrained region of *DSP*. Due to the vital function of desmoplakin in maintaining desmosomal integrity and anchoring intermediate filaments, truncating variants are anticipated to severely impair cellular adhesion in cardiac and epidermal tissues [41].

Eigen-PC: The raw rank score of 0.88297 obtained using Eigen-PC supports the deleterious effect of the variant. Eigen-PC is an unsupervised, integrative scoring system which combines functional genomic annotations and evolutionary constraints to create principal components. High Eigen-PC scores are characteristic of variants that are

subject to strong negative selection. The elevated score observed for p.Gln1433* is consistent with DSP's intolerance to LoF variants, thereby reinforcing the latter's role as a disease-causing mutation [42].

DANN: The DANN rank score of 0.87558 provides further support for pathogenicity. DANN uses deep neural networks to model the complex relationships between genomic features associated with deleterious variants. Scores approaching 1 indicate a strong predicted functional impact. The high DANN score for this nonsense variant is consistent with the predicted disruption to protein integrity and desmosomal function, providing further computational evidence of pathogenicity [43].

Integrated fitCons: The fitCons rank score of 0.72963 indicates that the affected nucleotide resides within a functionally constrained genomic region that is subject to purifying selection. fitCons estimates the probability that a genomic position is under selective pressure, using functional genomic data. The relatively high score suggests that variation at this site is poorly tolerated, which is consistent with the essential structural role of DSP in maintaining tissue integrity [44].

Likelihood ratio test (LRT): A converted rank score of 0.43081 from the LRT indicates moderate evolutionary constraint at the affected site. The LRT compares models of neutral evolution versus purifying selection across species. Although the score is lower than that of ensemble predictors, this is not unexpected for nonsense variants, since the LRT primarily evaluates conservation at the nucleotide level rather than the effects of protein truncation. However, it is important to note that the functional consequence of a premature stop codon outweighs modest conservation metrics, particularly in a gene with a well-established loss-of-function (LoF) disease mechanism [45]. Summary of in silico pathogenicity predictions for the DSP c.4297C>T (p.Gln1433) nonsense variant using multiple computational tools is presented in Table S1.

Table S1. Summarises the in silico analyses for *DSP*:c.4297C>T, showing the prediction tools that have the most significant pathogenicity scores.

**Algorithm / Tool**	**Score**	**Interpretation / Effect**
MutationTaster	1.0	Disease-causing; high-confidence pathogenic prediction
CADD	0.98556	Highly deleterious (top 1–2% of variants)
FATHMM-MKL (coding)	0.99201	Strongly pathogenic
Eigen-PC	0.88297	Strong negative selection; deleterious
DANN	0.87558	High functional impact
fitCons (integrated)	0.72963	Functionally constrained site
Likelihood Ratio Test (LRT)	0.43081	Moderate evolutionary constraint
